# Ocular and Systemic Risk Factors and Correlation with Glaucomatous Damage in Normal Tension Glaucoma

**DOI:** 10.7759/cureus.2638

**Published:** 2018-05-16

**Authors:** Vallinayagam Muthu Krishnan, Pandian Datta Gulnar, Rao Vasudev Anand, Chellappa Vijayakumar, Gopal Balasubramaniyan

**Affiliations:** 1 Ophthalmology, Sri Laksmi Narayana Institute of Medical Sciences, Puducherry, IND; 2 Ophthalmology, Sri Venkateswara Institute of Medical Sciences, Puducherry, IND; 3 Surgery, Jawaharlal Institute of Postgraduate Medical Education and Research (JIPMER), Puducherry, IND

**Keywords:** normal tension glaucoma, applanation tonometry, central corneal thickness, diastolic perfusion pressure

## Abstract

Background

Normal tension glaucoma (NTG) is a clinical entity that poses a diagnostic and therapeutic challenge. The study elaborates ocular and systemic risk factors of NTG in the South Indian population. It determines the correlation between risk factors and severity of glaucomatous damage.

Methods

This descriptive study was done on 81 eyes of 41 patients. A brief history of hypertension, heart disease, migraine, and family history of glaucoma were noted. The parameters measured include blood pressure, lipid profile, visual acuity, refraction, intraocular pressure (IOP) by applanation tonometry, diurnal variation, slit lamp biomicroscopy, central corneal thickness (CCT), and perimetry (Humphrey 24-2).

Results

The mean age was 51.75 years. There was female predilection (63.41%). Thirteen patients (32.5%) had hypertension, five (12.5%) had migraine, and seven had hyperlipidemia (17.5%). Perfusion pressure demonstrated a negative correlation value of -0.319 (rho value) with visual field defects (p<0.05) and -0.266 (rho value) with glaucomatous cupping (p=0.093). The IOP varied from 10 mmHg to 19 mmHg with a mean of 15.34. The average CCT was 522.06±36.09 microns. Neuroretinal rim thinning was seen in 12 eyes (14.8%), polar notching in six eyes (7.4%), and peripapillary atrophy in 20 eyes (24.6%). Two eyes (2.4%) had splinter hemorrhage at disc margin. A lower value of CCT was associated with lower IOP, a weak positive correlation (r value 0.121). Optic disc cupping is strongly associated with severity of field defects, r value 0.743, (p<0.00).

Conclusion

Normal tension glaucoma is common in females. Hypertension and lower diastolic perfusion pressure are important risk factors. Lower CCT is associated with lower IOP (applanation tonometry). Optic disc cupping and diastolic perfusion pressure strongly correlate with severity of visual field defects.

## Introduction

Glaucoma is the second common cause of blindness worldwide. One-third of patients with primary open angle glaucoma (POAG) are reclassified as normal tension glaucoma (NTG) [1]. NTG is a condition with an intraocular pressure of less than 21 mmHg on applanation tonometry, with characteristic optic disc cupping and visual field defects [2,3]. NTG is a clinical entity where substantial research is required to define patient characteristics and to correlate risk factors. This study throws light on the ocular and systemic risk factors of NTG in the South Indian population. It also determines the correlation between certain risk factors and the severity of glaucomatous damage.

## Materials and methods

A cross-sectional study was done on 41 outpatients (81 eyes) attending the glaucoma clinic for two years at a tertiary care hospital. Ethical clearance was obtained from the institutional ethics committee. Informed consent was obtained from all patients.

The inclusion criteria were a mean intraocular pressure (IOP) ≤ 21 mmHg (Goldmann applanation tonometry) on diurnal testing, open angles on gonioscopy, typical optic disc changes, and characteristic visual field defects. The exclusion criteria were patients with secondary cause for optic neuropathy (angle recession glaucoma, steroid induced glaucoma, and inflammatory glaucoma), corneal opacity, previous intraocular surgery, contact lens wearers, corneal edema, corneal astigmatism >2D and those with other optic nerve abnormalities or intracranial disease.

Preliminary demographic data of patients were noted. A brief history of hypertension, heart disease, haemodynamic crisis (acute blood loss following trauma, surgery etc.), family history of glaucoma, and migraine were noted. Blood pressure was measured and fasting lipid profile was done. The ocular examination included visual acuity (Snellen’s chart), refraction, tonometry (applanation), slit lamp examination, fundus examination (+90D/direct ophthalmoscopy), optic disc fundus photography, and CCT by ultrasonic pachymetry.

Optic disc cupping, neuroretinal rim thinning, disc hemorrhage, peripapillary atrophy, focal notching, nerve fiber layer defects, and laminar dot sign were examined. Patients were subjected to diurnal variation test. Field evaluation was done using Humphrey field analyzer (24-2). A second visual field report of every patient was taken for analysis accounting for consistency of findings and the patient’s learning curve. Visual field defects were analyzed using Anderson’s criteria and graded accordingly.

## Results

Eighty-one eyes of 41 patients were studied. The mean age in the study group was 51.75 [standard deviation (SD -10.91)] years. Twenty-six (63.41%) patients were females and 15 (36.59%) patients were male.

Systemic risk factors

**Figure 1:**
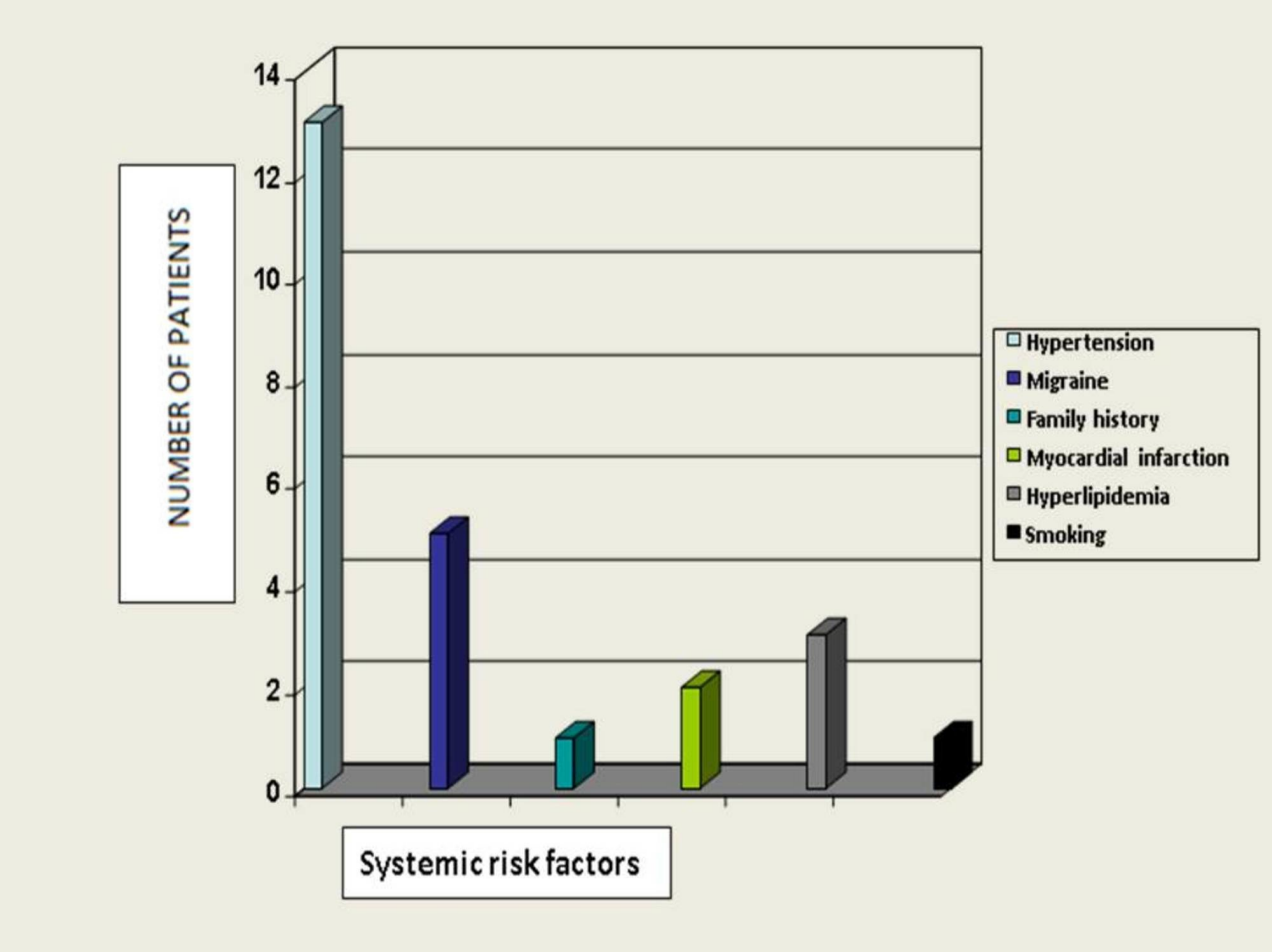
Distribution of systemic risk factors in study patients

Thirteen patients (32.5%) were hypertensive. Other risk factors were distributed as shown below (Figure 1).

**Figure 2:**
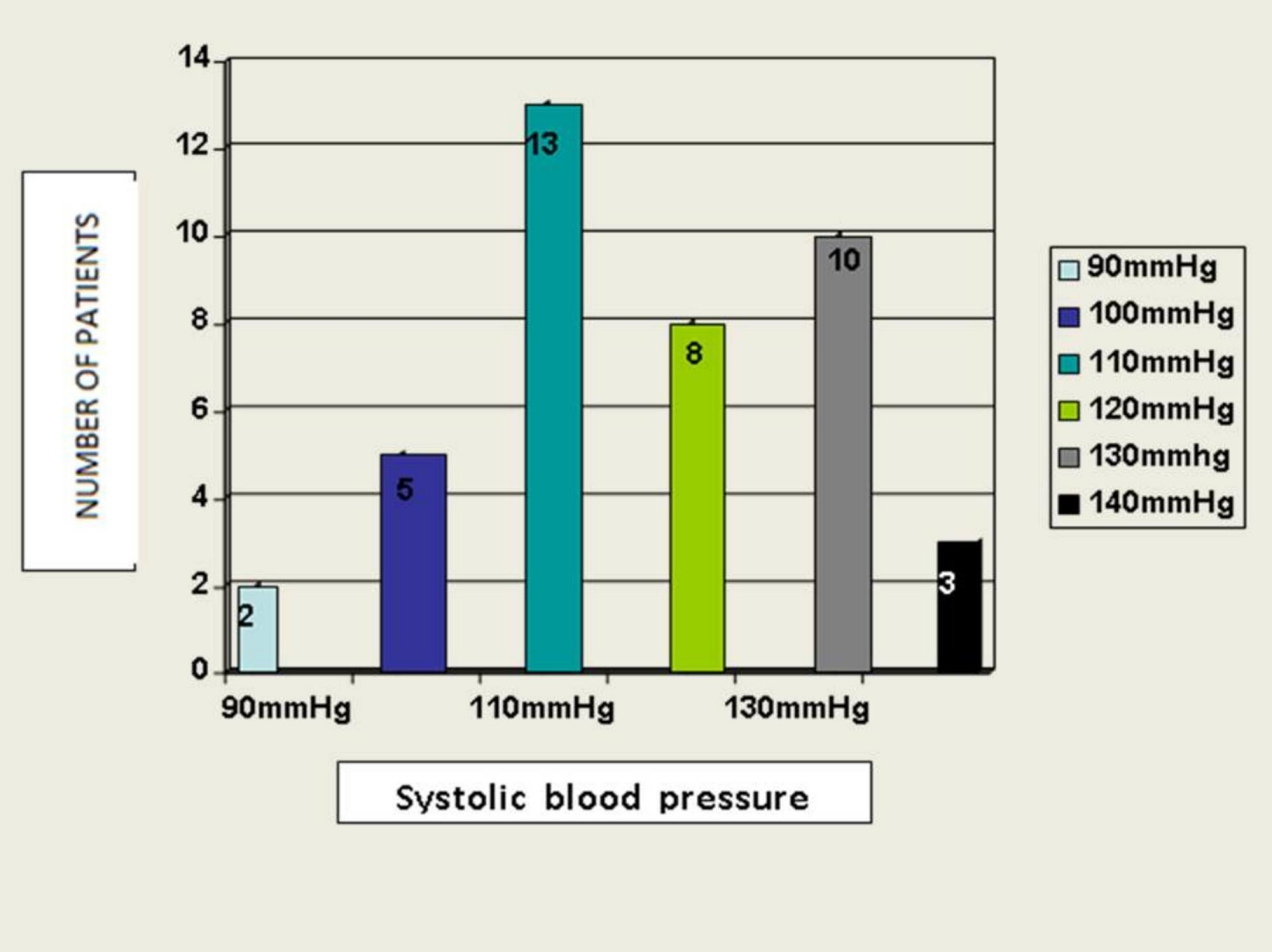
Distribution of systolic blood pressure in study patients

Twenty patients (48.78%) had systolic blood pressure ≤110 mmHg, and 21 (51.21%) patients >110 mmHg (Figure 2).

Perfusion pressure

**Figure 3:**
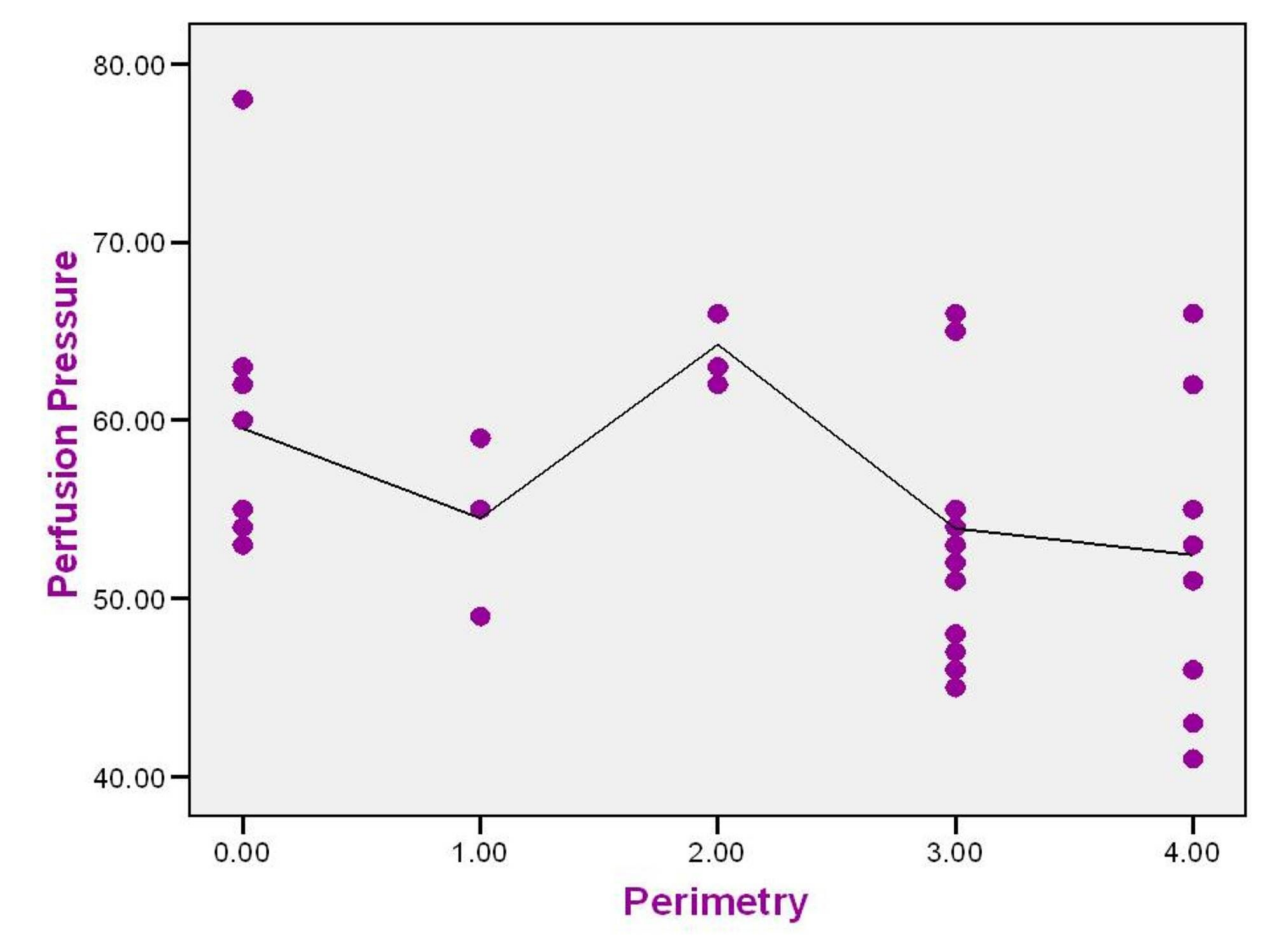
Correlation between perfusion pressure (mmHg) and visual field defect in study patients

Eight (20%) patients had ocular perfusion pressure (derived by subtracting intraocular pressure from diastolic blood pressure) below 50 mmHg. Perfusion pressure had a negative correlation (rho) value of -0.319 (p<0.05). The correlation is statistically significant and suggests worsening of visual field with decreasing perfusion pressure. Cup to disc ratio also had negative correlation with perfusion pressure, with rho value of -0.266 (p=0.093) (Figure 3).

Intraocular pressure (IOP)

The intraocular pressure (Goldmann applanation tonometry) varied from 10 mmHg to 19 mmHg with a mean IOP of 15.34 mmHg (±2.65 mmHg). Forty eyes (49.38%) had IOP >15 mmHg and 41 eyes (50.61%) <15 mmHg.

Central corneal thickness (CCT)

**Figure 4:**
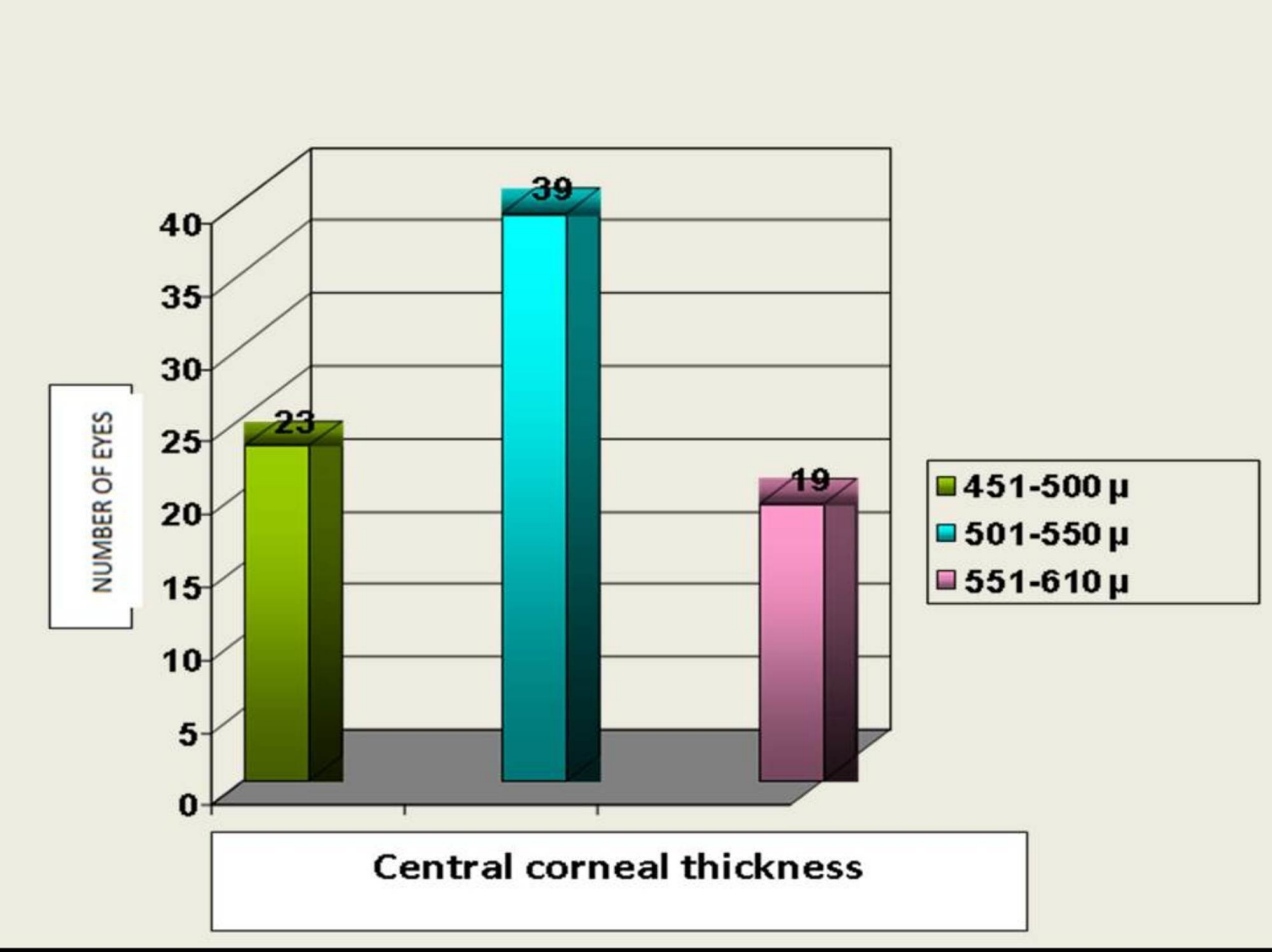
Distribution of central corneal thickness (microns) in study patients

About 50% eyes had CCT between 500 to 550 microns. Twenty-three eyes (28.39%) and 19 eyes (23.45%) had corneal thickness between 451 to 500 microns and 551 to 610 microns, respectively (Figure 4).

Cup to disc ratio

**Figure 5:**
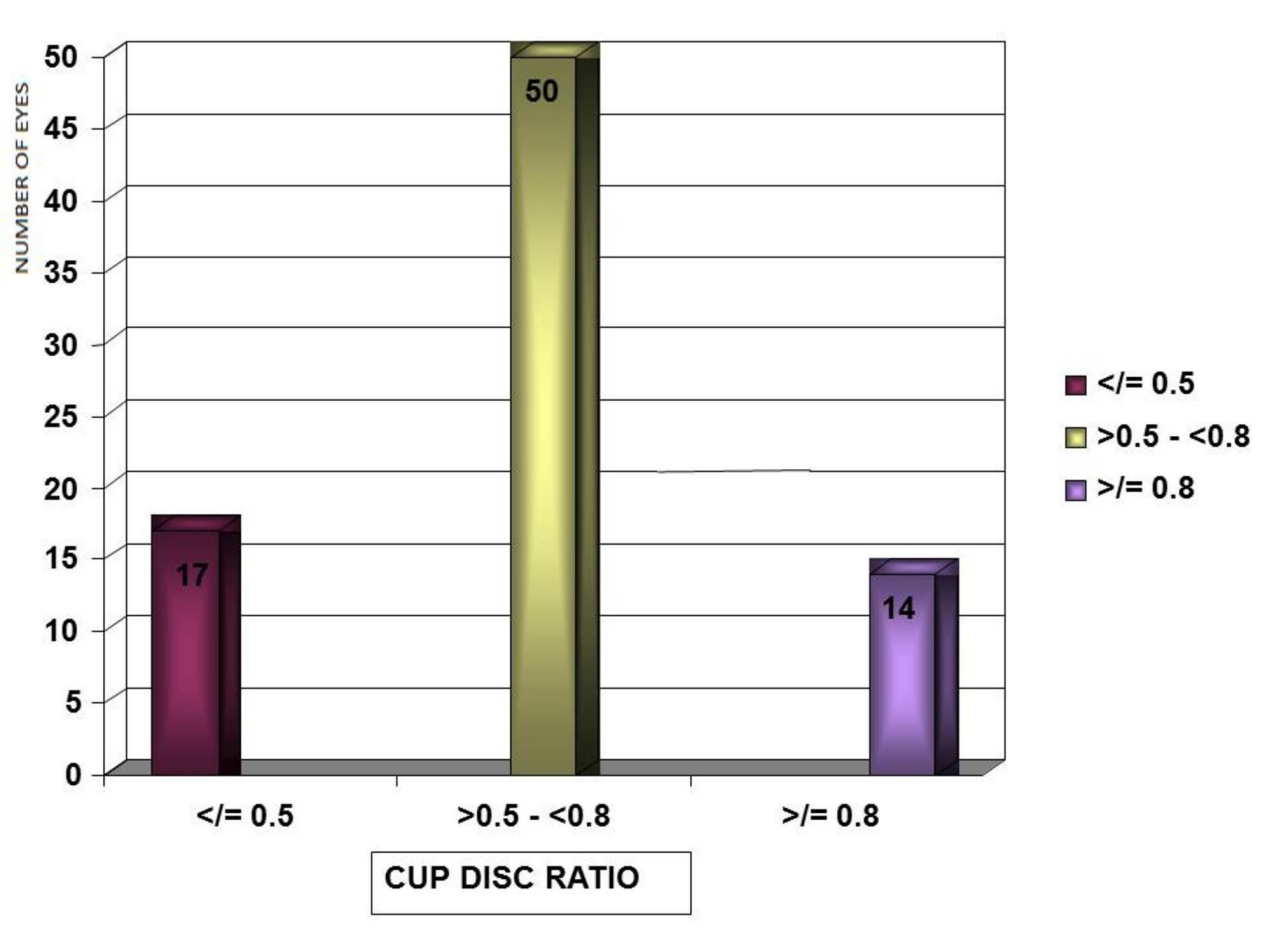
Distribution of cup disc ratio in study patients

Fifty eyes (61.7%) had a cup to disc ratio between 0.6 and 0.7. Seventeen eyes (20.9%) had a cup to disc ratio ≤ 0.5 and 14 eyes (17.2%) had a cup to disc ratio ≥ 0.8 (Figure 5).

Optic nerve head features

**Figure 6:**
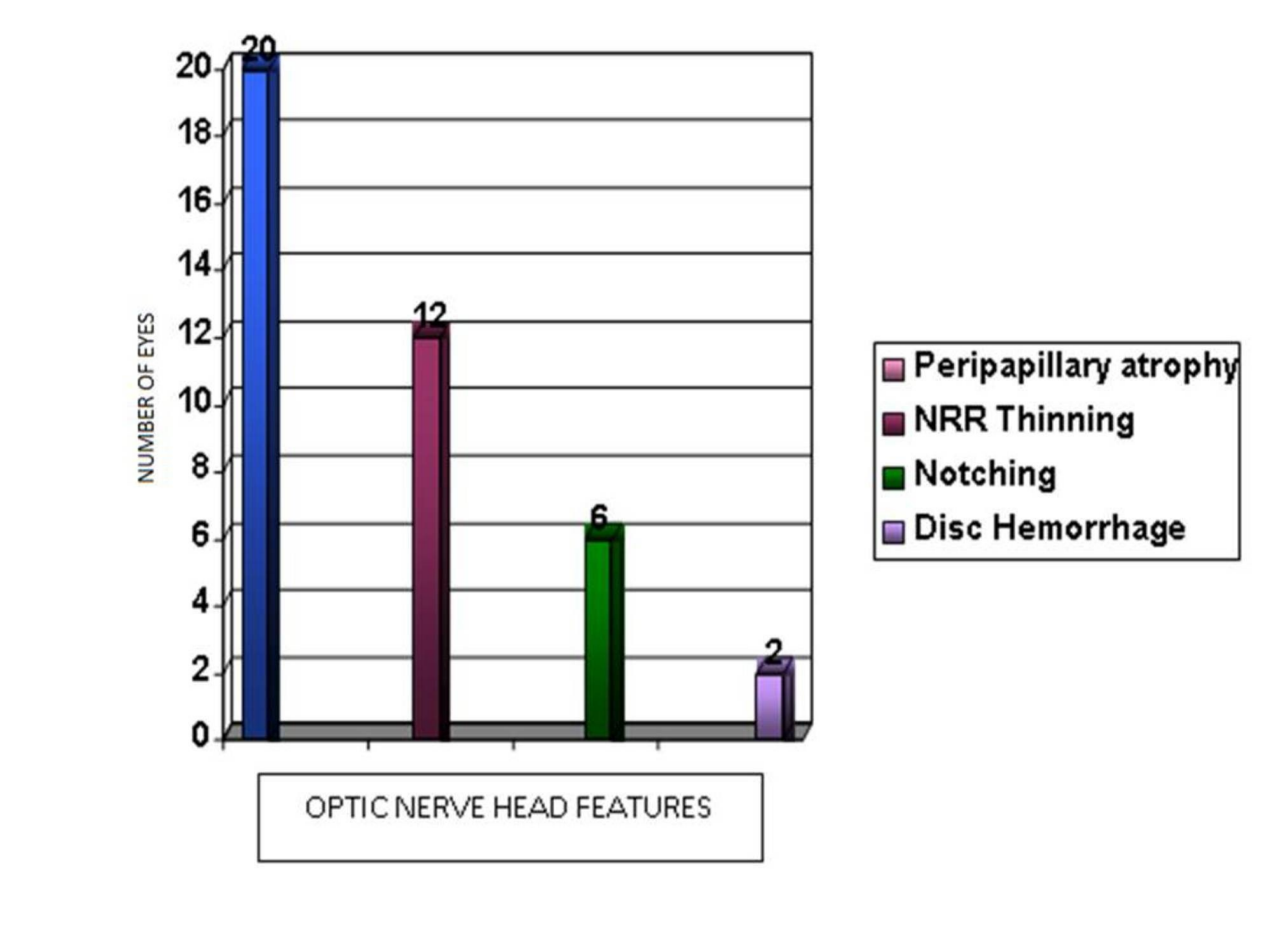
Distribution of optic nerve head features NRR: Neuroretinal rim

Neuroretinal rim thinning was seen in 12 eyes (14.8%). Six eyes (7.4%) had notching, and 20 eyes (24.6%) had peripapillary atrophy. Two eyes (2.4%) in this study had splinter hemorrhage at the disc margin (Figure 6).

Visual field defects

**Figure 7:**
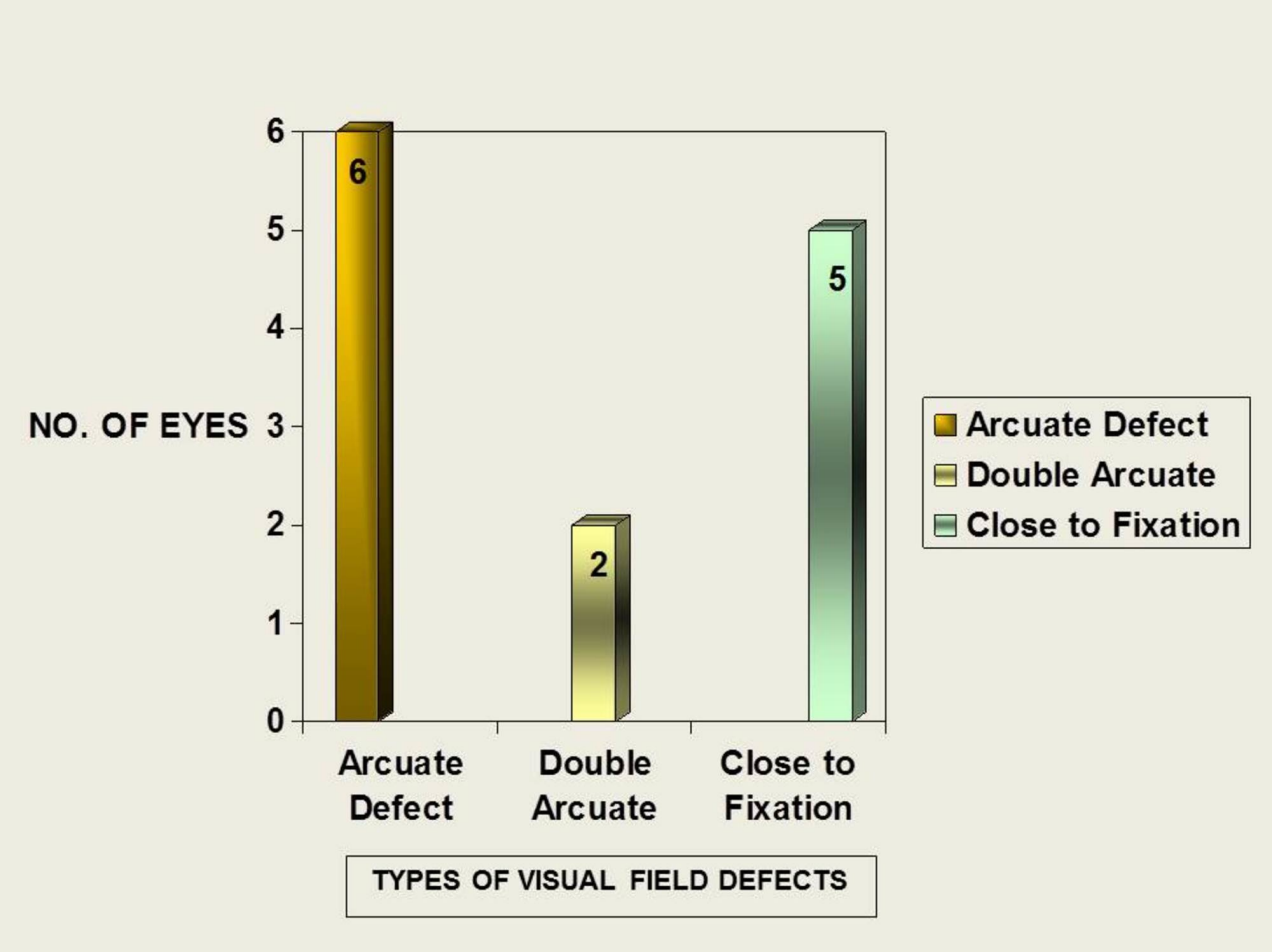
Distribution of types of field defects in study patients No: number

Twenty-one eyes (25.9%) had normal fields. Minimal and early field defects were seen in 10 (12.3%) and six eyes (7.4%), respectively. Moderate defects were seen in 27 eyes (33.3%), and severe field defect in 17 eyes (20.9%). Three eyes (3.7%) had field defects close to fixation. Localized defects were seen in four eyes (4.9%) with focal notching of neuroretinal rim. Arcuate defects were seen in six (7.4%) eyes of which three (3.7%) had superior arcuate and three (3.7%) had inferior arcuate defects. Double arcuate scotoma was seen in two (2.4%) patients. Twenty-eight eyes (34.5%) had field defects predominantly in the superior hemifield (Figure 7).

Correlation of central corneal thickness (CCT) and cup: disc ratio

**Figure 8:**
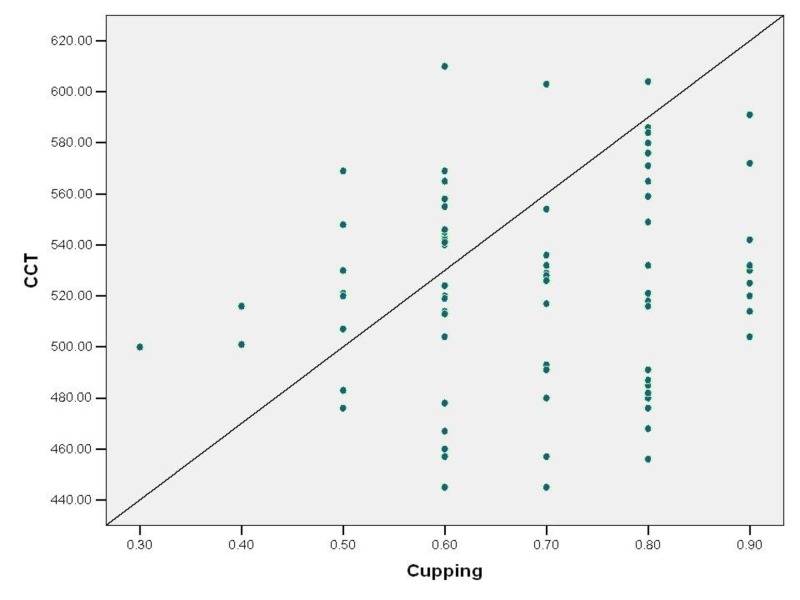
Correlation between central corneal thickness (microns) and cup:disc ratio in study patients CCT: Central corneal thickness

CCT and cup to disc ratio have a positive correlation with Pearson’s coefficient (“r” value) of 0.130. The correlation has no statistical significance as the p value is 0.248  (Figure 8).

Correlation of central corneal thickness (CCT) and intraocular pressure (IOP)

**Figure 9:**
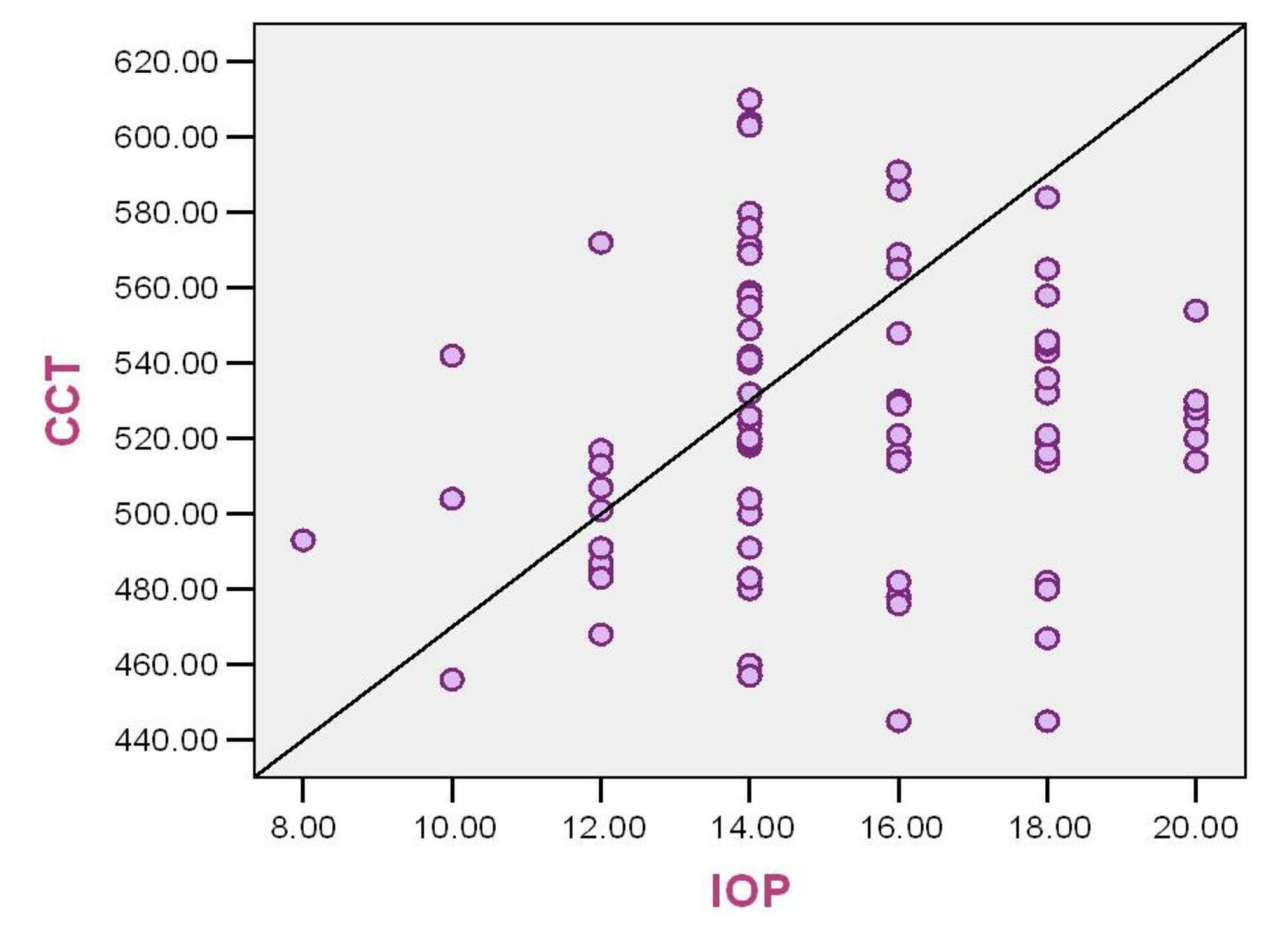
Correlation between central corneal thickness (microns) and intraocular pressure (mmHg) in study patients CCT: Central corneal thickness; IOP: Intraocular pressure

The measured IOP and CCT demonstrated a positive correlation. The Pearson’s correlation coefficient (r value) is 0.121. The level of significance (two tailed) is 0.282. With increasing CCT, there may be a false high measurement of intraocular pressure by Goldmann applanation tonometry (Figure 9).

Correlation of central corneal thickness and visual field defect

**Figure 10:**
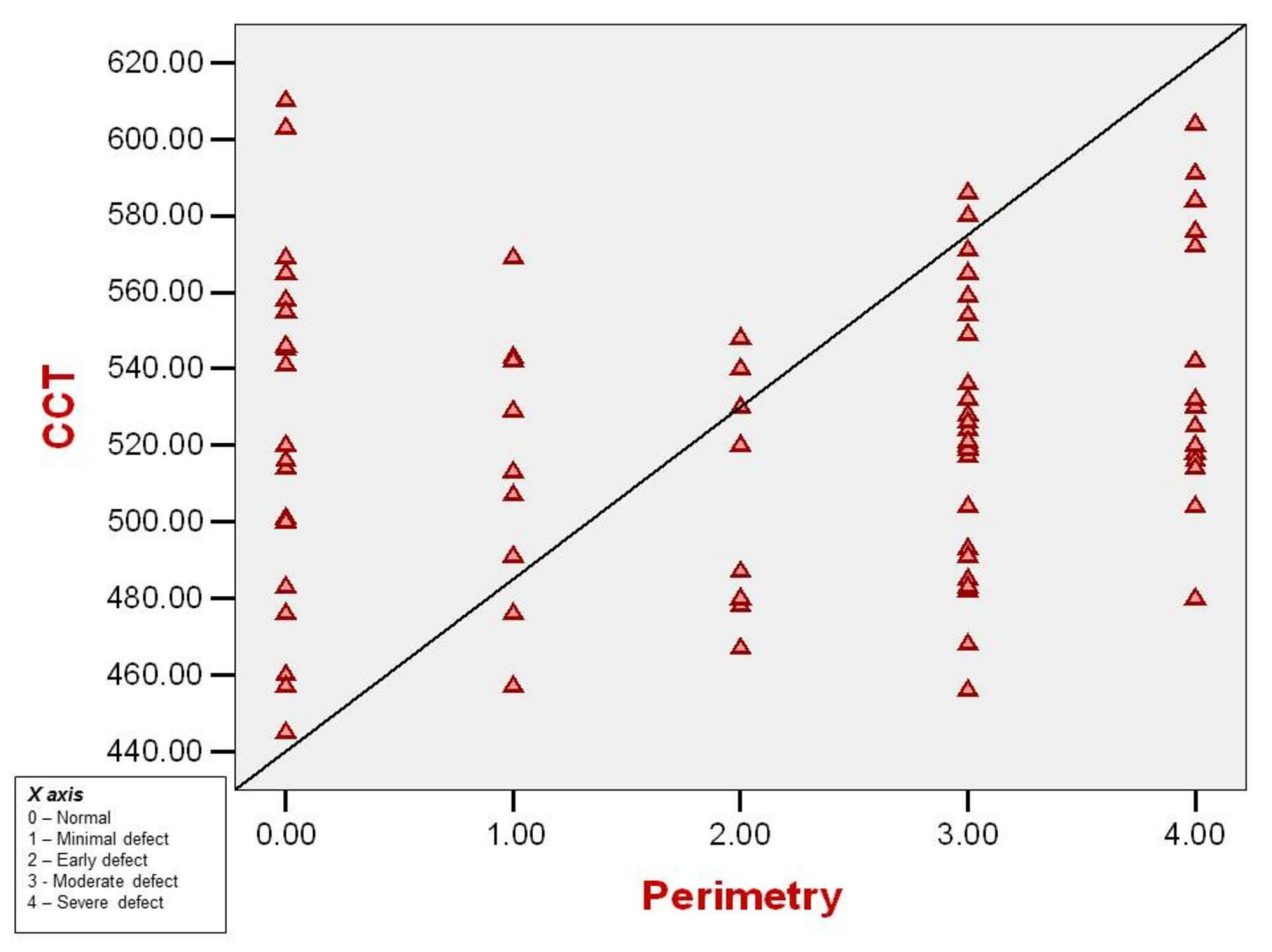
Correlation between central corneal thickness (microns) and visual field defect in study patients CCT: Central corneal thickness

CCT and visual field loss have a weak positive correlation by bivariate analysis with an ‘r’ value of 0.133 (Spearman’s rho coefficient) (Figure 10).

Correlation of cup: disc ratio and visual field defect

**Figure 11:**
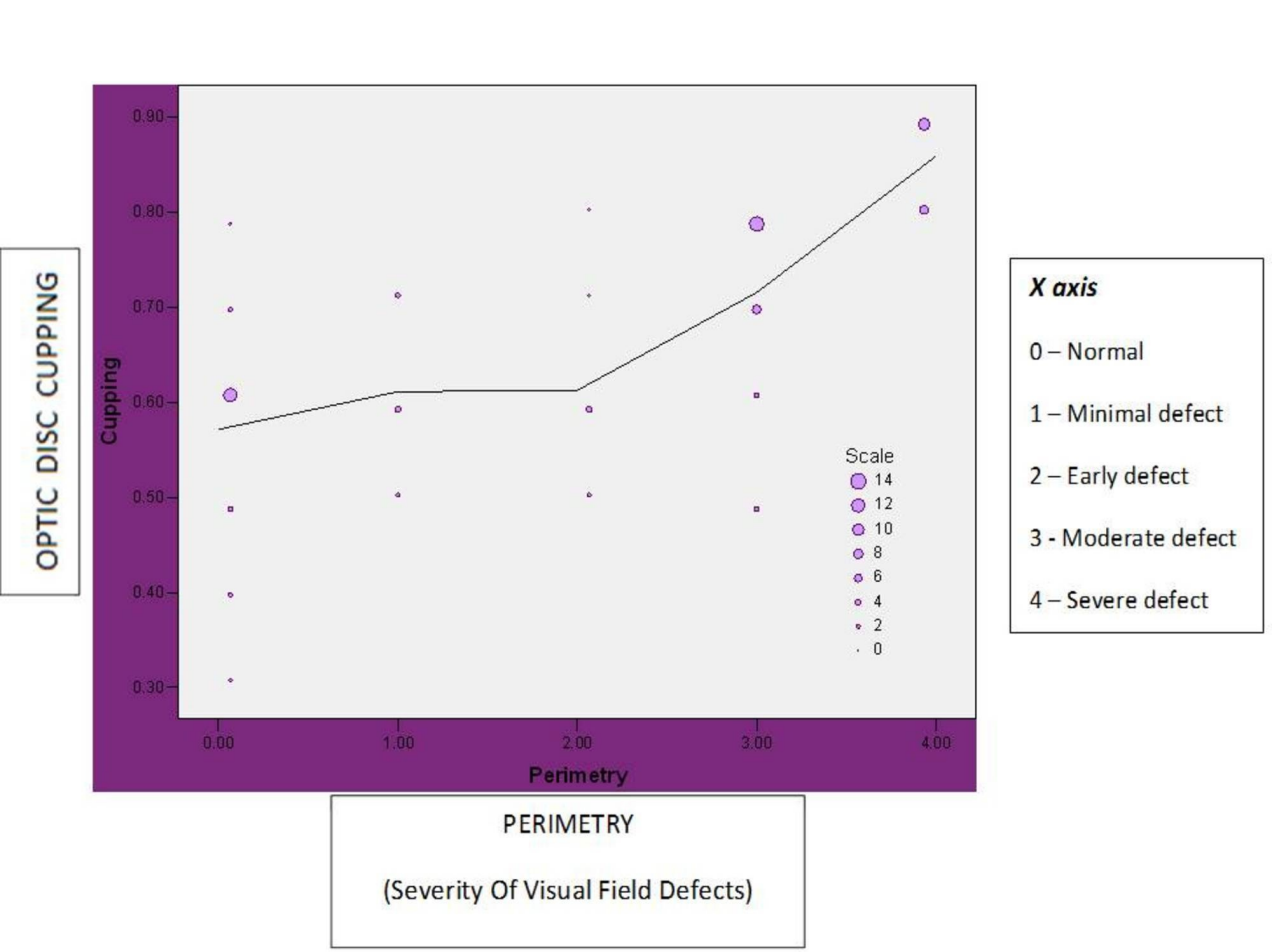
Correlation between cup : disc ratio and visual field defect in study patients

Cup to disc ratio has a strong positive correlation with visual field defect. The correlation coefficient “r value” is 0.743 (‘p’ <0.00). Hence the correlation is statistically significant (Figure 11).

Correlation between IOP, cup to disc ratio, CCT, and visual field defects

**Figure 12:**
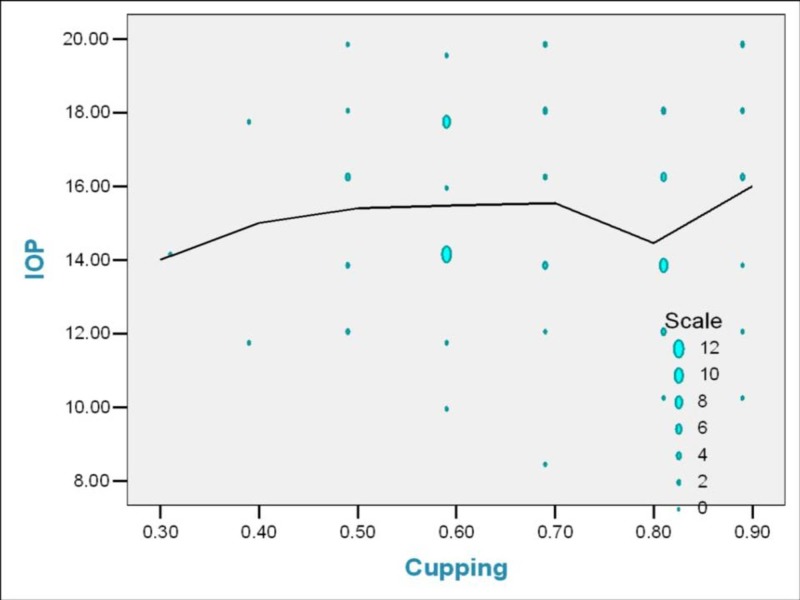
Correlation between intraocular pressure (mmHg) and optic disc cupping in study patients IOP: Intraocular pressure

**Figure 13:**
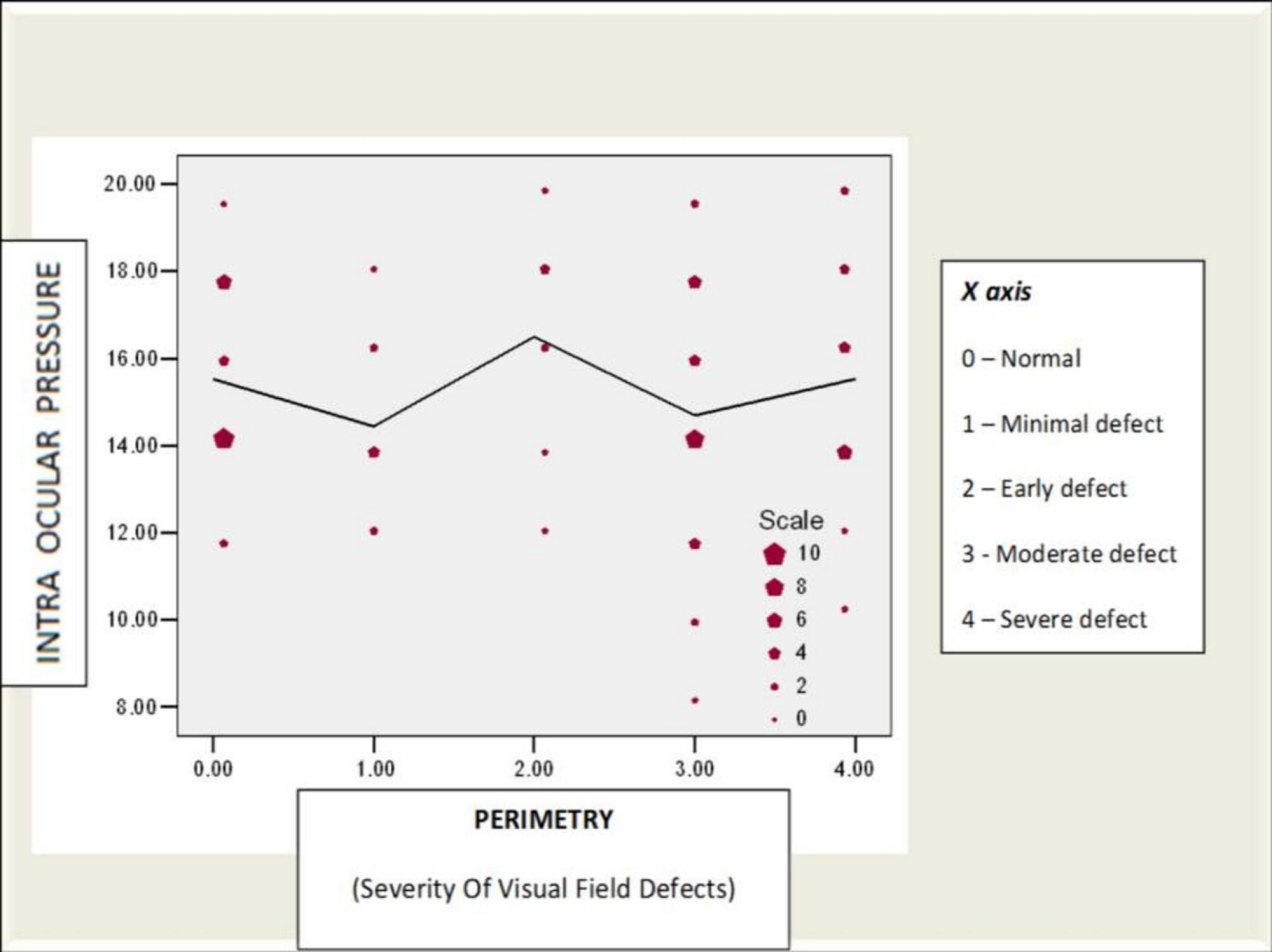
Correlation between intraocular pressure (mmHg) and field defect in study patients

The effect of IOP, cup to disc ratio, and CCT in predicting the severity of visual field loss is analyzed using linear regression analysis. The beta coefficient and level of significance are tabulated (Figures 12-13).

The most significant parameter affecting visual field defects is the severity of optic disc cupping. The β coefficient value is 0.703 with ‘p’ value of 0.000, suggesting that the visual field defect deteriorates with increasing cupping (Table 1).

**Table 1:** Correlation between intraocular pressure, cup disc ratio, central corneal thickness and visual field defect in study patients CCP: Central corneal thickness; IOP: Intraocular pressure

Parameters	Beta coefficient	‘p’ Value
IOP	-0.222	0.786
CCT	0.036	0.661
Cupping	0.703	0.000

## Discussion

Diagnosis of normal tension glaucoma (NTG) depends on ophthalmoscopic signs and field defects in the absence of other explanations for disc abnormality and visual field loss. This clinical entity poses a challenge for ophthalmologists and forces us to think about pressure dependent and pressure independent causal factors. The theories that explain the pathogenesis of NTG are mechanical and vascular theories, explained by structurally weak lamina cribrosa and vasospasm compromising optic nerve head perfusion. Vascular factors explain optic nerve head damage caused by migraine, Raynaud’s phenomenon, and smoking [4].

The collaborative normal tension glaucoma study group (CNTGS) has shown that intraocular pressure is a part of the pathogenic process of NTG [5]. Raised IOP is a modifiable risk factor that can be therapeutically manipulated [6]. The gold standard method for measuring intraocular pressure is Goldmann applanation tonometry (GAT), the accuracy of which is influenced by scleral and corneal rigidity, reflected by the CCT [7]. It has been proven that patients with NTG have reduced CCT and a thinner cornea is correlated with advanced disease [8,9]. The risk factors proposed in NTG were peripheral vascular disease, migraine, hypotension (nocturnal dips), hemodynamic crisis, and carotid insufficiency [4,6,10]. NTG has a variable course and hence it is important to determine the rate of progression before deciding appropriate therapy.

Age distribution

The mean age in the study group was 51.75 ± 10.91 years, which corresponds to the study done by Nana Meng et al. with a mean age of 51.9 ± 9.56 [6]. A study by Fang EN et al. had higher average age of 68.2 years [10]. Goldberg et al. reported an age range between 63 and 77 years [11].

Sex ratio

In this study, 63.41% of subjects were females, the ratio being 1.8:1 (female: male). Similar female preponderance was reported by Fang EN et al. (60%) and Goldberg et al. (63%, 2:1) [10,11].

Family history

In this study, positive family history was noted in one patient (2.5%). Zeiter JH et al. showed positive family history in 5-40% and Nana Meng et al. in 10% [6,12]. Goldberg et al. reported family history in 21% patients [11].

Systemic risk factors

Fang EN et al. found vascular risk factors to be significantly higher in patients with NTG and POAG [10]. Moreover, increase in risk factors was correlated with increase in progression of visual field defects. In this study, five (12.5%) patients had migraine and two (5%) had history of myocardial infarction. Goldberg et al. noted an increased prevalence of sedentary life style and cardiovascular disease in NTG [11]. Nana Meng et al. noted statistically significant influences of hypotension in 45%, migraine in 10%, and myocardial infarction in 5% of NTG [6].

In this study, diastolic perfusion pressure had an inverse correlation with visual field defect, which was statistically significant. Dada T et al. studied 113 eyes of NTG patients and proved that a larger circadian mean ocular perfusion pressure (MOPP) fluctuation was significantly associated with decreased mean deviation, increased pattern standard deviation, increased AGIS (Advanced Glaucoma Intervention Study) score and reduced neuroretinal rim area [13].

In the Baltimore Eye Survey, a diastolic perfusion pressure < 30 mmHg carried a six-fold higher risk. A diastolic perfusion pressure < 53 mmHg conferred a 2.2 times greater risk in the Barbados Eye Studies [4,14]. In the Barbados Eye Studies, risk factors for development of POAG were lower systolic blood pressure (relative risk = 0.91 per 10 mm Hg), systolic ocular perfusion pressure, diastolic ocular perfusion pressure, and mean ocular perfusion pressure [14].

The Early Manifest Glaucoma Trial showed cardiovascular risk factors to significantly affect long-term disease progression. Baseline predictive factors were lower ocular systolic perfusion pressure (≤160 mmHg), cardiovascular disease in patients with a higher baseline IOP, and lower systolic blood pressure (≤125 mmHg) in patients with lower baseline IOP [15].

Pillunat LE et al. hypothesized that generalized vascular disease may lead to NTG. He showed that there is lack of autoregulation in optic nerve head circulation only in NTG whereas Anderson suggested defective autoregulation in POAG and NTG [16].

In this study, seven patients (17.5%) had hyperlipidemia. Chumbley LC et al. reported hyperlipidemia in 41.5% NTG and 18% of POAG [17]. Goldberg et al. reported hyperlipidemia in 36%, which is higher than that reported by this study [11].

Hypotension aggravates visual field loss but hypertension may be protective till the onset of hypertensive vascular disease, which becomes a risk factor in its own right [18]. In this study, 45% of subjects had hypotension. Drance et al. reported hypotension in 31% patients [2]. Leighton et al. observed higher prevalence of hypotension in NTG [18]. Goldberg et al. found 21% of patients to have low systolic and 16% to have low diastolic blood pressure [11].

Treatment of hypertension, rather than the disease itself, is a risk factor for progression of glaucoma. Nocturnal dip in blood pressure augmented by nocturnal antihypertensives, is the major vascular risk factor defined [18]. In this study, 31.7% subjects had hypertension. The patients on antihypertensives with larger nocturnal dips had worse visual fields. Kamal D et al. found a lower nocturnal blood pressure correlating with progressive disease as compared to nonprogressive disease, in NTG and POAG. These studies provide a therapeutic option that nocturnal hypotension should be assessed in patients on antihypertensives, and therapy should be modified accordingly [19].

Disc hemorrhage is a vasospastic sign and is found to have a higher prevalence in NTG. This suggests that local circulatory changes within the optic nerve head are involved in the pathogenesis of NTG [20].

Optic disc cupping

Kamal D et al. performed an analysis on a group with NTG, POAG, and ocular hypertension to determine any significant disc morphology and found advanced neuroretinal rim loss and peripapillary atrophy in NTG. In this study, 12 (14.8%) patients had neuroretinal rim thinning. Acquired optic disc pit was more frequent in NTG [19]. Levene RZ et al. reported that the extent of cupping in NTG is often greater than would be expected from the size and depth of visual field defect [21]. This was supported by Caprioli J et al., who noted sloping cup margins and neuroretinal rim thinning in temporal and inferotemporal parts of the optic disc [22]. In this study, six patients (7.4%) had focal notching of disc, three superior and three inferior.

Caprioli J et al. showed that the degree of field defect is related to the peripapillary atrophy (especially beta zone) [22]. Twenty eyes (24.6%) of this study had peripapillary atrophy. Radcliffe NM et al. reported peripapillary atrophy in 84% [20].

Disc hemorrhage was observed in two patients (2.4%) in this study. Studies have reported disc hemorrhage to be more common in NTG. Drance et al. reported disc hemorrhage in 24.4% of NTG [2]. Levene et al. reported the same in 11.3% and 6.3%, respectively [21]. Kamal D et al. found that disc hemorrhage and β zone peripapillary atrophy were significantly more common in NTG [19]. The presence of disc hemorrhage at baseline increased the probability of neuroretinal rim loss by a factor of 5.56, compared to 2.19 in the group with elevated IOP [23].

Intraocular pressure (IOP)

Intraocular pressure is a risk factor linked with the development and progression of glaucoma. This modifiable risk factor, if reduced to lower levels, provides us with a therapeutic option.

Cartwright MJ et al. analyzed 14 cases of NTG with asymmetrical IOP and found that the eye with higher IOP suffered more severe glaucomatous cupping and visual field loss [24]. Though this study found an inverse correlation of IOP with visual field, the correlation may be due to chance (p value – 0.915). Similar to this study, Zeiter JH et al. showed that there was no relation between higher IOP and severe visual field loss in 78% of untreated NTG patients [12].

Kamal D et al. showed that an average reduction from 18 to 10 mmHg correlated with a significant slowing of progressive visual field loss [19].

The Normal Tension Glaucoma Study investigated the effect of 30% reduction of intraocular pressure on the rate of visual field progression. Zeiter JH et al. showed that a 25% lowering of IOP is essential to prove effective in slowing the rate of field loss [12].

Central corneal thickness (CCT)

The mean CCT (CCT) in this study is 522.06 ± 39.06 microns. This is lower than the average CCT of 534 (±30 microns) in glaucoma patients reported by Thomas R et al. He reported an average CCT in NTG in the South Indian population as 517 microns, which was slightly lower than the study group [8].

Ventura ACS et al. found the mean CCT to be thinner (518 ± 0.5) in NTG than in ocular hypertensives (563 ± 29), POAG (515 ± 35), and normals (524 ± 25) [7]. In a study by Copt R et al., CCT in NTG (521±31 microns) was significantly lower than that in the control group (552 ± 35 microns) and POAG (543 ± 35 microns), (p<0.001) [25].

The positive correlation between CCT and intraocular pressure has been well demonstrated in this study. The influence of scleral rigidity and CCT on intraocular pressure was first discussed by Goldmann and Schmidt. Copt R et al. postulated that Goldmann applanation tonometry yields accurate measurements only around 520 microns. The average error evoked by thicker or thinner cornea was 0.7 mmHg per 10 micron deviation from a “normal” value of 520 [25]. The intraocular pressure corrected for higher CCT resulted in corrected IOP of ≤ 21 mmHg in 39% of ocular hypertensives in a study by Thomas R et al. [8]. Lower CCT was significantly associated with worsened Advanced Glaucoma Intervention Study (AGIS) score, worsened mean deviation of visual field, increased vertical and horizontal cup-disc ratios, and increased number of glaucoma medications [9].

The Early Manifest Glaucoma Trial demonstrated that glaucoma progression was lessened by 10% for every mmHg decrease in IOP. So adjustments in intraocular pressure for decrease in CCT may alter a patient’s risk for visual field progression. POAG patients with thinner CCT may need closer follow-up and more aggressive treatment [5].

Visual field defects

Levene RZ et al. noted a higher frequency of dense defects extending to within five degrees of fixation in NTG (92%) than POAG (20%) [21]. In this study, there were five eyes (6.17%) with field defects close to fixation. This low prevalence may be due to the small sample size of this study. Caprioli J et al. showed that scotomas in NTG had a steeper slope, greater depth, and were closer to fixation [22].

In this study group, superior hemifield defects were seen in 28 eyes (34.5%). Chumbley LC et al. reported a higher percent of subjects (84%) with superior hemifield defects [17].

Kim JW et al. showed visual field progression in 53% at three years and 62% at five years, proving a linear mode of field progression in NTG [26]. Noureddin showed that progression occurred in 50% patients and 37% of eyes. No statistically significant difference was found between patients with progressive and non-progressive disease. The visual field defects were frequently located in the superior hemifield in both groups [27]. Fang EN et al. noted visual field progression in 34% POAG and 46% NTG [10].

## Conclusions

Normal tension glaucoma is more common in females. Systemic risk factors are closely associated with the severity and progression. A lower central corneal thickness is associated with lower intraocular pressure measurement on Goldmann applanation tonometry. Optic disc cupping and diastolic perfusion pressure correlate with the severity of visual field defects. Assessment of risk factors and determination of correlation of risk factors with glaucoma progression may provide us with a newer therapeutic strategy. NTG is a clinical entity where substantial research is indicated to define patient characteristics and to correlate risk factors with severity of glaucomatous damage.
